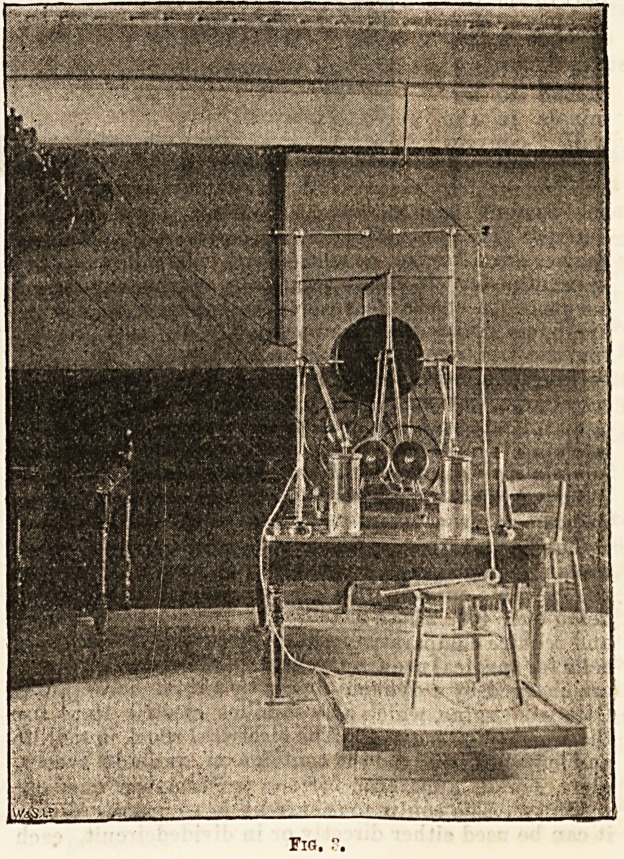# Some Simple Uses of Carbolic Acid

**Published:** 1893-07-08

**Authors:** A. G. Miller

**Affiliations:** Lecturer on Clinical Surgery, and Surgeon to the Edinburgh Royal Infirmary


FIG. 2.

Owing to the extensive use of electricity in this hospital the nurses receive special instructions in the manipulation of the various apparatus already described, and require to show proficiency in this special branch of medical and surgical treatment before receiving their certificates.

### SOME SIMPLE USES OF CARBOLIC ACID—II.

By A. G. MILLER, M.D., F.R.C.S.E., Lecturer on Clinical Surgery, and Surgeon to the Edinburgh Royal Infirmary.

Let me say a few words about these qualities of carbolic acid.

First, it is absorbable. This is easily demonstrated. If you put your finger into a solution of carbolic acid you will find that it penetrates the skin and permeates it so thoroughly that you are conscious of its presence in your skin for hours after by its peculiar sweet, pungent taste. But the rapid and perfect absorption of carbolic acid by the skin from a watery solution is best demonstrated by the fact of its appearance in the urine in many persons when the application is external and on a perfectly unbroken skin. Children seem to be peculiarly susceptible, possibly on account of their tender skins. It will be seen, therefore, that this substance must act very directly and decidedly on the skin on account of its absorbability, and will also act more or less directly on other tissues after it is absorbed.

Next consider the local effects produced by carbolic acid when it comes in contact with the skin or other tissues. The effect on the skin is very evident. If you put your hand in a strong solution of carbolic acid (say 1 to 20) the skin soon becomes white, wrinkled, and shrivelled. The hand feels easily chilled, and if the immersion be continued for some minutes, sensation is deadened, and the sense of touch is diminished. This is the astringent and anæsthetic action of the acid.

This action of carbolic acid I had an experience of long ago. In the early days of antiseptics I happened to cut my finger rather severely. I applied some carbolic lotion (it must have been 1 to 10), which caused me intense pain. This was followed quickly, however, by complete subsidence of all pain and sensitiveness of the wound. On describing this to my friend, Professor Rutherford, he told me that probably the carbolic acid acted by coagulating the exposed nerve filiments in the wound, and so making them incapable for the time of conducting sensation.

Lastly, I would refer to the antiseptic power of carbolic acid—its germicidal action. The estimate of this by scientific observers has varied considerably from time to time. But carbolic acid has always maintained a high position as a germicide and antiseptic, and has lately been brought again into prominent notice and favour by Sir Joseph Lister himself.

Now, taking for granted that you believe in the absorbability, the astringent and anæsthetic action, and the antiseptic power of carbolic acid, let me direct your attention to some ordinary and simple uses of the drug.

(*a*) It is the best application for making the hands aseptic—even antiseptic. This is apparent from its quick and thorough absorption. A watery solution easily parts with its carbolic acid to the skin, which greedily takes it up, and after a while passes it on to the deeper tissues and to the circulation. In this way the skin of the operator's hands is quickly purified, and remains for a considerable time carbolised. My own method of hand cleansing is as follows: I rub some soft soap into my hands thoroughly, sometimes with the addition of turpentine. I then use water, and wash my hands, and then steep them in carbolic lotion (1 to 40) for some minutes. In the course of my operations, after having carbolised my hands, I do not re-dip them in carbolic lotion on account of the anæsthetic action to which I have referred. I prefer to use corrosive lotion.

(*b*) Carbolic lotion is usually the best antiseptic to

FIG. 3.

employ for purifying the skin of a patient on whom an operation has to be performed. It has many advantages. The carbolic acid (aided by the water) softens the cuticle so thoroughly that after twelve, or, better, twenty-four hours' application of the carbolic towel, the cuticle can be readily removed by rubbing. The antiseptic also penetrates into the hair follicles and sweat glands, and so is peculiarly adapted for cleansing such parts as the pubes and axilla. Its anæsthetic action helps to make the skin less sensitive, and a slight operation may be performed by its aid alone. For this purpose a concentrated solution may be employed—say, 1 in 10. Some employ a solution in glycerine, but this is not so good as a watery solution, for glycerine does not part with carbolic acid so easily as water does.

If the carbolic acid be absorbed too readily, and it shows its usual reaction in the urine, no harm will be done provided the application is at once stopped, and some other antiseptic, such as corrosive lotion, employed. I have never seen symptoms of dangerous poisoning from an external application.

(c) Carbolic acid is eminently adapted for the treatment of simple or septic inflammations of the skin. These are due generally to the presence of micro-organisms, and the carbolic acid being a germicide "goes for" them, and, still further, being readily absorbed, gets at them quickly and directly. Then the pain, heat, redness, and swelling of the inflammatory process being due to vascular congestion mainly, are at once and directly antagonised and subdued by the anæsthetic and astringent action of the carbolic acid.

This sounds very theoretical, but practically it is as satisfactory as theoretically it seems neat. I have proved to myself and others frequently that carbolic lotion will check and resolve an inflammatory process far quicker and more thoroughly than any simple fomentation or other application could. It ranks, therefore, as what the older surgeons used to call an antiphlogistic of great power and efficacy.

Many years ago it was recommended in the treatment of erythema and erysipelas. I remember, some twenty years ago I daresay, sponging an erythema with carbolic lotion (1 to 40), and seeing it fade distinctly, but only to revive again. I did not think then of a constant application. I think that carbolic lotion ought to be useful in some forms of erysipelas, but I cannot speak definitely, for I have had no opportunity of giving it an extended trial.

(d) Chronic ulcers are greatly benefited by a thorough soaking with carbolic lotion. Pain, swelling, and congestion are diminished, and the offensive odour of the discharge may be completely removed. I have seen a healthy action started by a few days of the "carbolic towel."

(e) Varicose veins also are often much benefited, especially if inflamed.

(f) Deep inflammations even, such as synovitis and periostitis, are influenced, for, as we have seen, carbolic acid can penetrate to the deeper structures. The effect, is not so rapid or powerful, however, as can be easily imagined.

(g) I do not know a better treatment for a septic puncture, or hospital pustule on the finger, than a good soaking with (1 to 20) carbolic lotion renewed twice daily. I have seen many threatened bad fingers in my residents, students, nurses, and myself get quite well in a day or two without suppuration, and others that were treated too late to prevent suppuration greatly benefited. I could go on enumerating circumstances in which carbolic acid is useful, sprains and bruises for instance, but I shall refer specially to only one more.

When the carbolic spray was dispensed with in my wards one of my nurses told me she was very sorry, because she could always cure a cold in her head by inhaling the spray.

## Figures and Tables

**Fig. 3. f1:**